# Trash-Talking and Trolling

**DOI:** 10.1007/s12110-018-9317-3

**Published:** 2018-05-26

**Authors:** Kevin M. Kniffin, Dylan Palacio

**Affiliations:** 000000041936877X grid.5386.8 S. C. Johnson College of Business, Dyson School of Applied Economics and Management, Cornell University, Warren Hall 111, Ithaca, NY 14853 USA

**Keywords:** Rivalry, Sports, Gossip, Insults, Communication, Evolutionary anthropology

## Abstract

Among the extra-physical aspects of team sports, the ways in which players talk to each other are among the more colorful but understudied dimensions of competition. To contribute an empirical basis for examining the nature of “trash talk,” we present the results of a study of 291 varsity athletes who compete in the top division among US universities. Based on a preliminary review of trash-talk topics among student-athletes, we asked participants to indicate the frequency with which they have communicated or heard others talk about opposing players’ athleticism, playing ability, physical appearance, boyfriends, girlfriends, sexual behavior, parents, and home institution during competitions. Our three main findings are: (1) Trash-talking is most commonly about the proximately important topic of playing ability while ultimately relevant topics such as physical appearance also appear to be common; (2) Men appear to trash-talk significantly more than women, and consistently across topics; and (3) contact sports such as football, hockey, lacrosse, and wrestling are associated with trash talk significantly more than other sports. We also examined whether the anonymity provided by face-masked helmets in “combat sports” was associated with more trash talk than contact sports played without a helmet (e.g., wrestling) and found no consistent association with face masks. Our findings highlight the ways in which competitors in physical sporting contests attempt to use language—often in ways that focus on players’ kin or reproductive interests—in pursuit of victory while establishing a baseline for future research into trash-talking.

Much of the activity that comprises an athletic contest does not involve physical activity but, instead, features communication among players. North American football games, for example, typically run more than 360 min from start to finish whereas the “game clock” runs for 60 min and actual play occupies approximately 11 min (Biderman 2010). Although in some sports (e.g., soccer), play is more continuous because there are fewer stoppages, team-level contests typically involve at least one intermission or half-time where teammates and coaches can talk to each other and, more basically, most sports usually provide opportunities for competitors to exchange words.

Prior research on communication among teammates has shown—sensibly—that it tends to be beneficial for players to engage each other both verbally and nonverbally during games (e.g., Kraus et al. 2010); however, communication between players who are competing against each other has not been studied as closely. Nevertheless, consistent with the fact that sporting events either produce winners and losers or, at least, rank-orderings among the contestants, it is understandable that verbal exchanges during competitions are often reported to be antagonistic. Indeed, certain players (e.g., Muhammad Ali) often acquire reputations for being especially verbose with respect to “trash-talking” their opponents during competition (e.g., Albom 2009; Zirin 2005). Academic attention to trash-talk has been sufficiently sparse, though, that Yip et al. (2018:129) describe trash-talking as “a novel construct in the organizational behavior literature” that their work on the relationships between trash talk, creativity, and ethical behavior “introduce[s]” (2018:139).

Regarding the theoretical vantage points from which to examine trash-talking, which is defined by Yip et al. as “boastful comments about the self or insulting comments about an opponent that are delivered by a competitor typically before or during a competition” (2018:126), there is ample reason to consider evolutionary perspectives just as in other forms of (physical) aggression (e.g., McDonald et al. 2012; Van Vugt 2012). Specifically, given that trash-talking is a kind of verbal aggression (e.g., Rainey 2012) meant to establish higher standing (and, perhaps, fitness) in relation to rivals, it is worth examining the degree to which it reflects underlying evolutionary dynamics. Similarly, the variant of trash-talking known as *trolling*, whereby rivals typically have no physical interaction, warrants investigation to examine the possible relevance of selective pressures. Specifically, in light of research on the prosocial behaviors that are cultivated when people are not anonymous (e.g., Zhong et al. 2010), it is possible that when interactions are mediated and partially anonymized through some kind of technology (e.g., face masks) people will be more likely to be hurtful to competitors.

Within the framework of Tinbergen’s (1963) four questions (as reviewed by Mishra 2017), the question of “why” trash-talking appears to be a persistent aspect of competitive team sports should be approached in a variety of ways. Is trash-talking just literal noise? Or, perhaps trash-talking was selected across evolutionary time because its benefits outweigh the potential costs given the possibility that some rivals might derive extra motivation from a trash-talker? In a recent application of an evolutionary approach to a common but understudied aspect of competitive sports, a study of tennis players found that lower-frequency grunting tended to be associated with winning (even early on in matches) and, more generally, established numerous baselines for future research into the nature of grunting (Raine et al. 2017). Although grunting is less actively aggressive toward a rival or set of rivals than trash-talking, both can fit the definition of intergroup aggression proposed by Manson and Wrangham (1991) that lists “threatening” as the first of several activities that qualify as “aggression.”

In this article, we examine trash talk in the context of contemporary sports before presenting the results of a field study we conducted in order to better understand the nature of trash-talking among actual players. Both our review and discussion will draw on and apply concepts from a variety of disciplines, with the goal of providing a more integrated or synthesized view. The three main dimensions on which our analysis will focus are (1) whether in-competition trash-talking is about topics unrelated to performance as much as it is about performance; (2) the degree to which there are sex differences in trash-talking (e.g., perhaps trash-talking is largely an example of intrasexual selection among men; Puts 2010); and (3) whether and how trash-talking patterns vary across sports. The exploratory and descriptive nature of our study provides a foundation for future research.

## Trash-Talking and Disciplinary Traditions

Even though it is a common topic among sports journalists and others, including fans, Yip et al. (2018) understandably described themselves as “introduc[ing]” the topic academically since (*a*) in the broad scope of research on human nature, “trash-talking” is relatively novel as a label for certain types of communication, and (*b*) prior studies on trash-talking have been conducted outside of the fields from which they draw. With respect to the categorical question, our search of the Human Relations Area Files (HRAF) for any mention of “trash talk” unsurprisingly yielded no findings since the category is both relatively novel and colloquial. On the other hand, we are confident that elements of trash-talking are likely found frequently in traditional anthropological reports by one or more different labels. Toward that end, similar to the value gained by adding color to imagery that was originally available only in black-and-white (e.g., Dirnhuber 2018), we embrace and apply Sherry’s (1995) anthropology-based call for researchers to “ancestralize” our perception of modern groups while concurrently “modernizing” our perception of ancestral groups. In this respect, our exploration of trash-talking in contemporary sports environments is also responsive to calls (e.g., Roberts et al. 2012) to apply evolutionary thinking to problems that have organizational relevance (e.g., Kniffin 2009).

Rather than reinventing the wheel, we accept Yip et al.’s (2018) definition of trash talk and can add to their characterization that it tends to be adversarial and often apocryphal in ways that tend to be hyperbolic and/or are intended to be humorous. Given these descriptors, the similarities between trash-talking and certain kinds of gossip are strikingly clear. Notwithstanding differences in how people classify gossip, we can draw on Kniffin and Wilson’s (2005) description of gossip as “informal evaluative talk” about others who are or are not present and, specifically, the subcategory of “negative personal talk” as an abbreviated descriptor for trash-talk.

Although outside of the scope of our current study, we can observe that negative gossip often tends to be adversarial and/or apocryphal or humorous (from the perspective of the speaker). For example, in a study of competitive intercollegiate rowers, when a teammate was not pulling his weight during various preparations for the season, teammates took it upon themselves to engage in “negative personal talk” about the slacker in ways that were adversarial, apocryphal, and (from the perspective of the speakers) humorous (Kniffin and Wilson 2005, 2010). Whereas trash talk is typically about an individual’s opponents and gossip is often about fellow members of an individual’s team or organization, value is nevertheless gained by recognizing that at least one of the ways in which “trash talk” would have been discussed in the traditional anthropological record would be in the context of reporting about “gossip.” Indeed, this is especially true when one accepts a broader definition of gossip that includes statements made about others who are not necessarily part of one’s community (see De Backer et al. 2016; Dunbar 1998; McAndrew and Milenkovic 2002; McAndrew et al. 2007; Scalise Sugiyama 2016 for more evolutionary analyses of gossip).

Yip et al. (2018) do break important ground with respect to the implications of trash-talking, but it is an overstatement that they “provide the first conceptual definition of trash-talking” since a limited number of previous studies from other disciplines examine the topic. For example, the *Journal of Sport Behavior* has published work on “trash talk” (Rainey and Granito 2010; Rainey 2012); however, it is reasonable to expect that the disciplinary “distance” (Kniffin and Hanks 2017) between “sport management” and other fields accounts for the independent examination that each authorship group brought to the topic. Regardless, we are interested to integrate work from across disciplines for the sake of broader and more holistic analyses.

## Research Questions

Three research questions (RQs) serve as the framework for our study, relating to (1) the nature of trash talk, (2) the degree to which there are sex differences in trash-talking, and (3) whether trash-talk patterns differ across types of sports, with specific attention to (a) contact versus noncontact sports and (b) combat sports in which players do or do not wear (anonymizing) face masks. Given the relative dearth of prior research and the absence of prior work informed by evolutionary perspectives, our investigations are exploratory and have the goal of providing baselines with which future research can conduct more specific hypothesis-testing. The first of our three questions regarding the nature of trash-talking offers a prime illustration of both the novelty of considering trash talk in an evolutionary light and the value gained by initiating basic research on the topic.

As an example of trash talk from outside of sports, the verbal attacks made by President Donald Trump when he was campaigning for his party’s nomination illustrate the fact that trash-talking often involves banter about an opponent in relation to traits of evolutionary significance. As reviewed by Hall et al. (2016), Trump publicly celebrated the relative size of his hands compared with those of his final opponent for the party nomination, explicitly suggesting that their hand-sizes reflected the size of their respective reproductive organs. Of course, the size of one’s reproductive organs—or hands, for that matter—should have no bearing on one’s qualifications to serve as president; however, it was and remains remarkable that the winning candidate engaged in trash talk about such a topic with apparent success rather than, say, limiting strong and aggressive rhetoric to matters of public policy on which the candidates might thoughtfully disagree.

In the context of sports, trash-talking during competitions is typically not directly accessible to regular fans or observers; instead, we tend to learn about its frequency and possible impacts through autobiographical and journalistic accounts (e.g., Albom 2009). In perhaps the most high-profile example of trash-talking in team sports that reached the level of general visibility, the star player in a men’s World Cup soccer final near the end of the match was ejected after headbutting a player who, as reviewed by Rainey and Granito (2010), had been insulting the star’s mother and sister. As with the example provided by Trump in which trash-talking concerned reproductive organs rather than policy, it is remarkable from an evolutionary perspective that banter intended to unsettle a player would involve kin rather than one’s abilities as a soccer player. Indeed, the two examples are complementary given the evolutionary relevance of reproduction and genetic relatedness.

Our interest to explore the nature of trash talk in more detail was prompted partly by accounts we have heard from high-level competitors who themselves have heard other players talking about opponents’ romantic partners, siblings, and parents. Indeed, we heard about more than one intercollegiate team whose non-starting players had been unofficially assigned to scout upcoming opponents’ social media profiles in order to gain potential leverage. Given that information and communication about such topics should have no relevance for a person’s athletic performance, yet nevertheless is an activity that some players feel compelled to undertake, we set out to examine systematically the degree to which trash-talking relates differentially (or not) to proximately- or ultimately-related topics.

- **RQ1:** Is the content of trash talk differentially about topics of proximate or ultimate evolutionary significance?

Whereas RQ1 is designed to explore the nature of trash talk in relation to evolutionary questions of individual and inclusive fitness, RQ2 examines the degree to which sex differences might exist with respect to trash-talking. Given the central focus that sex differences have typically played in evolutionary analyses (e.g., Cronin 1993; Wilson 1994), it is sensible to consider whether men and women vary with respect to the frequency and/or content of trash-talking during sports. Taking the axiomatic sex difference that undergirds much of evolutionary psychology, whereby the potential reproductive variance for men is far higher than for women (e.g., Buss 1995), it is imaginable that trash-talking in contests involving men would be more frequent and more reproductively focused than trash-talking in contests involving women; however, there has not been prior research into this topic.

Indeed, although the content of trash-talking bears similarities to the negative personal talk reported in gossip research, the activity of trash-talking is worth recognizing as a kind of verbal aggression. In turn, once one appreciates that trash-talking entails a kind of aggression, it is valuable to recognize that there is—thanks largely to the work of evolutionary psychologists motived by sexual selection theory—ample evidence that men tend to demonstrate more aggression than women, both verbally and physically (e.g., Buss and Perry 1992; Campbell 2006; Geary 2010) as measured in real-world (Archer 2004) and laboratory (Bettencourt and Kernahan 1997) settings.

On the specific topic of sex differences in sports, previous research has traditionally presumed that “boys and men . . . possess a greater motivational predisposition to be interested in sports, especially team sports” (Deaner et al. 2012). Deaner et al. (2016) more specifically contend that it is differential interest rather than disparate opportunities that account for differences in playing patterns. Applying the concept of leks in a way that presumes that team sports function partly as a means for individuals to display their relative fitness, Apostolou and Lambrianou (2017) highlight ways in which sports are understood to be a means for people, especially men, to show off. As a test of the robustness of this view, Balish et al. (2016) present comparable patterns of differential sex-based interest in sports across a variety of cultural contexts.

For a finer-grained understanding of sex differences in competiveness, Apicella et al. (2017) report a replication of an overall sex difference but find no sex difference when examining interest in competition against oneself. In other words, they report that women tend to show a generally lower level of interest in competition, and they also find that women show comparably high levels of interest to compete against their own records. Although team sports are inherently about competing against others, the fact that sports tend to be structured in sex-specific ways, whereby teams composed exclusively of either men or women tend to play each other, Apicella et al.’s findings suggest that women (because they are playing against women) might show comparable levels of trash-talk—notwithstanding the more traditional sexual selection framework that presumes that men will display more aggressiveness or combativeness.

- **RQ2:** Do the frequency and content of trash-talk vary as a function of sex?

Independent of sex differences that might exist with respect to trash-talking in sports, it is also worthwhile to consider whether there are differences across sports given the wide variety of activities that fall under the label. For example, is bowling, table tennis, or golf a sport in the same way as soccer, basketball, and ice hockey? It is outside of the scope of our interests to focus on defining which activities qualify as a sport—for example, some might consider the trading of insults to be a sport or game (e.g., Bruhn and Murray 1985; Guest 2014); however, it is nevertheless useful for the purpose of establishing baselines to examine the degree to which trash-talking might vary across types of sports.

The two dimensions that we explore involve (a) whether a sport includes contact between contestants and (b) whether contestants wear a mask for their face. The first dimension recognizes the substantial differences across sports with respect to how much contact is involved. In a recent review, Fair and Champa (2017) use injury data to classify a set of 12 men’s intercollegiate sports and find that contact sports have overall injury rates that are—on average—two or more times higher than comparable rates for noncontact sports. Although Fair and Champa build on their observations to estimate the costs that are incurred (e.g., for medical care) as a result of injuries associated with contact sports, their findings are notable in relation to our interests since it is reasonable to expect that sports that involve more contact will also involve more trash-talking among contestants given the direct and immediate physical contact that players initiate and encounter.

The second dimension—wearing a face mask (from within the subsample of combat sports)—allows us to consider the degree to which the anonymity found in certain sports might encourage more trash-talking among contestants. From this perspective, it is worthwhile to examine whether the anonymity provided by face masks tends to invite a kind of “troll”-like trash-talking wherein players might feel less accountable for verbal aggression vis-à-vis opponents. Indeed, experimental research (Cheng et al. 2017) suggests that environmental conditions (e.g., the frequency of other trolling comments on a given online thread) can be modified to encourage online “trolling” in ways that would appear less involved than asking someone to wear a helmet and face mask. Fortunately for the sake of our investigation, in combat sports such as wrestling and rugby, contestants do not wear face masks; consequently, we can explore that distinction among combat sports with respect to the frequency and content of trash talk.

Notwithstanding finer-grained categorizations of sports, it is interesting to recognize that contact sports would more closely facilitate the “rehearsal” of “coalitional intergroup aggression” that Scalise Sugiyama et al. (2018) have highlighted in their evolutionary analysis of team sport in general. It is against such a backdrop that evolutionary studies on the importance of intergroup conflict in human evolution (e.g., McDonald et al. 2012; Van Vugt 2012) would also invite the question of whether trash-talking is the verbal component of a de facto package of activities that is bundled within contact sports more than, say, sports such as tennis or golf.

- **RQ3:** Do the frequency and content of trash talk vary across sports, with specific attention given to whether sports involve contact and whether players (within combat sports) wear face masks?

## Method

291 undergraduate student-athletes (141 men) from a private university competing in the National Collegiate Athletic Association’s (NCAA) top division in the northeastern United States participated in this survey-based study. Participants’ average age was 20 years (*SD* = 1.2 years).

With Institutional Review Board approval, a lottery drawing for three main prizes served as the participation incentive. The prizes were gift cards worth $75, $150, and $300.

Participants were invited from the full set of varsity teams at the university, with variable levels of active recruiting supplemented by individual coaches or related administrative staff. Sports represented by at least 10 participants were (in alphabetical order): gymnastics, ice hockey, lacrosse, rowing, soccer, squash, swimming and diving, track and field, volleyball, and wrestling.

Via an online survey over a span of six weeks during a recent spring semester (2016), participants responded to a survey that asked them (*a*) a number of demographic questions (e.g., on which teams they belonged) and (*b*) a pair of questions that asked them (1) “How often you’ve ever engaged in trash-talking against opponents” and (2) “how often you’ve ever seen others engage in trash-talking” in relation to a set of eight different topics, which were generated from anecdotal reports and were presented in randomized order. Subjects were asked to rate frequency on a scale of 1 (not at all) to 7 (very frequently).

Participants used this 7-point scale to indicate how frequently either they or others have communicated in a game setting regarding eight different topics (listed alphabetically here): athleticism, boyfriends, girlfriends, other team’s institution, parents, physical appearance, playing ability, and sexual behavior. For each topic for each question (regarding their own or others’ trash-talking), participants were also invited but not required to provide an example of the topic-specific trash-talking. Illustrative examples of trash-talking (by category) include “You’re a scrub” and “Does your coach know you’re out here [on the playing field]?” (Playing Ability); telling opponents they attend a “safety school” that is presumed to have lower standards for admission (Institution); and calling opponents “fat” or “ugly” or making fun of an opponent’s hairstyle (Physical Appearance).

Analyses were conducted using SPSS Version 24, and a dummy variable was created to indicate whether respondents participated in a contact or noncontact sport. Respondents in contact sports played (in alphabetical order with number of respondents in parentheses) baseball (6), basketball (5), fencing (3), field hockey (3), football (8), ice hockey (19), lacrosse (22), polo (9), soccer (29), softball (5), squash (12), and wrestling (23); respondents in noncontact sports consisted of equestrian (9), gymnastics (11), rowing (56), sailing (7), swimming and diving (28), tennis (7), track and field (22), and volleyball (7). The category of contact sports includes activities in which physical contact among contestants is routine (e.g., in squash, it is common for players in the relatively small confines of their courts to be penalized for interference or physical blocking of their opponents).

To examine whether face masks have an impact on trash-talking, we conservatively delimited the scope of comparison so that only “combat sports” were being compared: wrestling (no face mask) versus football, hockey, lacrosse, and “sprint football” (a variant of football formerly recognized as “lightweight” since players are limited to relatively low weights). There are admittedly different ways whereby one can categorize these sports (e.g., Deaner and Smith 2013); however, it seemed reasonable—for purposes of considering the effect of a face mask as an anonymizer—not to consider squash, baseball, soccer, or polo since there would seem to be markedly less aggression in those sports.

## Results

As illustrated through the basic descriptive statistics reported in Table 1 (focusing on the first data column), we can see—in relation to RQ1—that Playing Ability is the most commonly reported topic of trash-talking for both self- and other-reported ratings. Table 1 also shows that players tend to engage more distant (rather than game-specific) topics with frequencies that are comparable to those of more proximately connected topics. For example, Physical Appearance (PhysAp) is rated with relatively high frequency throughout Table 1 and when we compared—for the full sample—the average Physical Appearance and Athleticism (Ath) ratings, we found no significant difference for other-reported ratings (*M*_PhysAp_ = 3.72 ± 1.97 [*SD*], M_Ath_ = 3.70 ± 1.98, *t* = .27, *df* = 290, *p* = .79), although Athleticism did appear more frequently for self-reported trash-talking than Physical Appearance (M_PhysAp_ = 2.65 ± 1.84, M_Ath_ = 3.06 ± 2.00, *t* = 4.25, *df* = 290, *p* < .001).

**Table 1:** Reported occurrence (*M, SD*) of trash-talking topics in full sample and by sex

	Full Sample (*N* = 291)	Men (*N* = 141)	Women (*N* = 150)	*F*
Self-reported				
Playing Ability	3.44 (2.10)	3.96 (2.24)	2.95 (1.83)	17.82***
Athleticism	3.06 (2.00)	3.52 (2.08)	2.63 (1.83)	14.91***
Girlfriends	1.57 (1.30)	1.93 (1.59)	1.24 (.83)	21.84***
Boyfriends	1.40 (1.11)	1.48 (1.26)	1.33 (.95)	1.19
Physical Appearance	2.65 (1.84)	3.04 (1.96)	2.29 (1.66)	12.48***
Sexual Behavior	1.68 (1.41)	2.08 (1.70)	1.30 (.92)	24.00***
Parents	1.50 (1.16)	1.79 (1.47)	1.22 (.66)	18.79***
Institution	3.12 (1.89)	3.09 (1.85)	3.16 (1.93)	.10
Other-reported				
Playing Ability	4.14 (1.97)	4.45 (1.97)	3.86 (1.94)	6.57*
Athleticism	3.70 (1.98)	3.90 (2.02)	3.51 (1.92)	2.90
Girlfriends	2.23 (1.75)	2.79 (1.94)	1.71 (1.35)	31.13***
Boyfriends	1.70 (1.27)	1.73 (1.34)	1.67 (1.20)	.15
Physical Appearance	3.72 (1.97)	4.06 (1.97)	3.40 (1.91)	8.50**
Sexual Behavior	2.26 (1.74)	2.77 (1.94)	1.79 (1.36)	24.98***
Parents	1.97 (1.45)	2.25 (1.63)	1.70 (1.21)	10.70***
Institution	3.87 (1.90)	3.87 (1.90)	3.87 (1.94)	0.00

* *p* < .05, ** *p* < .01, *** *p* < .001

A more general observation to make from Table 1 is that—as expected—whether due to response bias whereby respondents were reluctant to admit to engaging in certain kinds of trash-talking directly or to actual patterns, there is a tendency for the self-reported trash-talking frequencies to be significantly lower than those for other-reported responses. Indeed, when we tested for the significance of differences for each topic of trash-talking within the full sample, we uniformly found a significant difference with *p* < .001 for all eight topics. Since Physical Appearance is one of the trash-talking topics of ultimate significance for which respondents might have felt a bias to under-report for themselves, it is interesting that it is the variable most different for self- versus other-reported ratings, whereby the difference in means is 1.07 compared with the next-largest difference of .75 for institution-focused trash-talking. Separate from response bias, though, it is also notable that self-reported communication is limited to a single person’s communications whereas other-reported communications are based, particularly for team sports, on communications from many others.

As for RQ2 and sex differences, Table 1 also presents the results of a full-sample comparison for men and women with respect to each of the trash-talking ratings. Each of the 11 contrasts for which there was a significant difference involved topics for which ratings for men were higher than those for women. When the pattern of ratings for self- and other-reported trash talk is examined (Table 2), note that there are more sex differences for self- than other-reported trash-talking. Such a pattern may be due to women feeling more response-sensitivity on these questions; however, it is outside of the scope of the current study to examine the pattern further.

**Table 2:** Reported occurrence (*M, SD*) of trash-talking topics in contact and noncontact sports (*N* = 291)

	Contact Sports	Noncontact Sports	*F*
Self-reported			
Playing Ability	4.15 (2.05)	2.76 (1.92)	35.70***
Athleticism	3.60 (2.01)	2.54 (1.86)	21.92***
Girlfriends	1.90 (1.59)	1.25 (.82)	19.36***
Boyfriends	1.53 (1.36)	1.28 (.78)	3.68
Physical Appearance	3.34 (1.95)	1.97 (1.44)	46.35***
Sexual Behavior	2.02 (1.67)	1.34 (.99)	18.04***
Parents	1.78 (1.48)	1.22 (.62)	17.37***
Institution	3.30 (1.92)	2.95 (1.84)	2.56
Other-reported			
Playing Ability	4.72 (1.85)	3.59 (1.93)	26.00***
Athleticism	4.08 (1.98)	3.32 (1.91)	11.21**
Girlfriends	2.81 (2.00)	1.67 (1.22)	35.00***
Boyfriends	1.88 (1.44)	1.52 (1.04)	5.92*
Physical Appearance	4.35 (1.80)	3.10 (1.93)	32.75***
Sexual Behavior	2.85 (1.93)	1.69 (1.30)	36.36***
Parents	2.38 (1.62)	1.56 (1.14)	24.48***
Institution	4.19 (1.83)	3.56 (1.96)	7.86*

* *p* < .05, ** *p* < .01, *** *p* < .001

In regard to RQ3, Table 2 indicates that trash-talking does appear to be significantly more common in contact sports than noncontact sports. In fact, the pattern of contrast shows significantly higher frequencies for six of the eight topics for self-reported responses and all of the topics for other-reported ratings. When we conduct the analyses separately for men and women, a robust pattern is visible for men, whereas differences among women are limited. Although suggestive of an interaction effect between whether a sport involves contact and the sex of the respondent, closer exploration of such interactions for the gamut of trash-talk topics that we are examining here is beyond the scope of our present study.

With regard to combat sports in which players’ faces are covered (football, ice hockey, and lacrosse) or uncovered (wrestling), we focused our analyses on men since the numbers of women in our sample were not comparable, largely because—there were no women’s teams for football or wrestling, and face masks are not worn in women’s lacrosse. As indicated in Table 3, there is no consistent tendency for differences in the frequency of topics between combat sports that do or do not entail players wearing a face mask. Of the two differences that are significant, one fits the expectation that players will be more aggressive behind a mask whereas the other runs in the opposite direction.

**Table 3:** Reported occurrence (*M, SD*) of trash-talking topics in combat sports played with and without a face mask

	With Face Mask (*N* = 32)	Without Face Mask (*N* = 23)	*F*
Self-reported			
Playing Ability	5.56 (1.22)	3.70 (2.14)	16.84***
Athleticism	4.59 (1.78)	2.63 (1.83)	1.91
Girlfriends	2.38 (1.76)	2.17 (1.70)	.18
Boyfriends	1.13 (.42)	2.09 (1.95)	7.35**
Physical Appearance	4.16 (1.80)	3.48 (2.15)	.77
Sexual Behavior	2.28 (1.78)	2.57 (2.04)	.30
Parents	1.66 (1.13)	2.35 (2.23)	2.29
Institution	3.31 (1.93)	3.04 (2.03)	.25
Other-reported			
Playing Ability	5.56 (1.66)	4.70 (1.66)	3.63
Athleticism	4.91 (1.96)	4.22 (2.00)	1.63
Girlfriends	3.66 (2.09)	3.70 (2.01)	.01
Boyfriends	1.69 (1.49)	2.17 (1.53)	1.40
Physical Appearance	5.03 (1.60)	4.96 (1.58)	.03
Sexual Behavior	3.25 (2.11)	3.74 (2.34)	.66
Parents	2.63 (1.91)	3.09 (1.83)	.81
Institution	4.28 (1.63)	3.91 (2.00)	.57

* *p* < .05, ** *p* < .01, *** *p* < .001

## Discussion

Prior research on trash-talking has been sufficiently scant that our study was motivated by research questions as a means for guiding exploratory analyses. In terms of our research questions, we find that: (1) while the most common trash-talking topic is playing ability, there is a substantial amount of trash-talking on topics related to ultimate fitness, such as physical appearance; (2) men trash-talk significantly more than women; (3a) there is more trash-talking in contact sports than noncontact sports, and (3b) whether or not a player’s face is masked from their opponent does not appear to increase the frequency of trash-talking within the subsample of combat sports.

With regard to our first finding, it is sensible that trash-talking in sports would focus most strongly and consistently on the topic that is most pertinent to competing in sports—playing ability. On the other hand, the relatively high frequency of topics with more ultimate relevance such as physical appearance lends credence to the view that important themes in the content of trash-talking exist, especially given research conducted by evolutionists finding that attractive appearance (e.g., Sugiyama 2005) and perhaps even athleticism (e.g., Fink et al. 2016) can be reflective of “good genes.” Indeed, although the dichotomy between proximate and ultimate is useful for distinguishing the types of trash talk, it also reflects a difference of degree rather than a difference in kind since disparagement of a person’s playing ability, for example, is presumably intended to reflect a person’s fitness. Future research that examines whether trash-talking about distantly related topics such as physical appearance also exists at different levels of sport such as high school or professional ranks would be helpful. Among professional athletes, it is plausible—in light of work showing that competitiveness appears to dip after marriage (e.g., Farrelly and Nettle 2007)—that older players trash-talk less about distantly related topics than on topics such as playing ability, and perhaps older or married players trash-talk less about all topics. From the other end of the age spectrum, it would be interesting to examine the degree to which trash-talk potentially precedes or coincides with hormonal changes associated with adolescence. More broadly, it would be interesting in the context of intergroup conflict to understand the degree to which trash-talking might be accompanied by changes in how members of a given out-group are perceived by in-group members given prior work that has shown such patterns in relation to political parties and their respective leaders (Kniffin et al. 2014).

With regard to our second finding, the present study yields robust evidence—complementing prior research on other forms of aggression (e.g., Geary 2010)—that trash-talking appears to be more commonly undertaken by men than women, suggesting that trash-talking is reflective of intrasexual selection among men (see Puts 2010 for a review of similar domains). Future research that examines masculinity and femininity in addition to sex would be helpful in light of prior research showing that the two dimensions of sex and masculinity/femininity are not isomorphic (e.g., Spisak et al. 2012); perhaps masculine males and females are more likely to trash-talk than feminine females and males. Similarly, although men tend to be larger than women, it would be worthwhile to consider the degree to which physical size (or formidability) might be more explanatory than sex for at least certain kinds of trash-talking. In addition, future research that considers athletes’ 2D:4D ratio as a low-cost marker of testosterone differences (e.g., Stenstrom et al. 2011) could be helpful for understanding any biological or hormonal connection with trash-talking. Of course, as with each of our findings, it would also be helpful to consider these questions across culture groups to look for variation in the content and/or frequency of trash-talking. For example, with respect to sex differences, it seems plausible that countries or communities with highly egalitarian sex roles might show less difference than we found in our sample. Lastly, although all of the sports that we examined in our study are either men’s or women’s teams, the growing trend toward coed athletic contests (e.g., Costa 2018) obviously invites further consideration with respect to the frequency and any possible effects of trash-talking.

With regard to our third finding, it is sensible to expect—given the wide variation in the nature of activities that are recognized commonly as sports (e.g., chess is sometimes recognized as a sport in major publications such as *Sports Illustrated*)— varying frequencies of trash-talking across sports, especially with respect to whether a sport entails aggressiveness or physical contact between contestants. The general finding that we report, whereby contact sports—among men, at least—appear to involve significantly more trash-talking than noncontact sports, is consistent with the view that trash-talking is a form of aggressive communication. Our lack of finding an effect of having one’s face masked suggests that anonymity does not increase the frequency of trash-talking among competitors. Future research would be valuable that draws on different samples so comparisons can be examined for women as well as men and—with respect to the finer-grained question of whether face masks matter within combat sports—a broader sampling of players who compete in non-masked sports (e.g., rugby). Future research should also consider the fact that face masks are not the only means of anonymity in a competition. For example, players in online eSports competitions can be anonymous in relation to their competitors, and there is ample evidence that trolling occurs among eSports contestants, often with very adverse consequences (e.g., Blackburn and Kwak 2014).

More basic limitations of the current findings center largely on the fact that our data are based on reporting rather than direct observations. Although our collection of self- and other-reported trash-talking data offers a better chance to consider the robustness of our findings than if we had only measured one of the two perspectives, it would be ideal if future research were able to collect direct observational data via technology that would allow for unobtrusive listening to players during games or matches. There are undoubtedly challenges to collecting such data (e.g., players are routinely facing and speaking in different directions), but it nonetheless is one way to improve upon the type of data that we employed for the current study. Given the proclivity for professional athletes to use social media (e.g., Filo et al. 2015), it is also imaginable that analyses of public posts would provide another helpful source of data. In this vein, it is interesting to recognize the possibility that the growth of social media has perhaps greatly increased the frequency of trash-talking in sports since rivals are now better able and more likely to find personal information about each other in advance of directly competing as athletes share more about themselves online (perhaps partly with the goal of building their own marketable brands; Tiago et al. 2016).

With regard to measures that were not recorded through our survey instrument, we can highlight that future research would be needed to examine whether superstar players, for example, are more likely to trash-talk—perhaps because it serves as a reinforcing signal of their dominance. Similarly, anecdotal reports (e.g., Bishop 2009) suggest that players who were raised with older siblings might be more likely to have sharpened their verbal jousting skills as they grew up. With respect to additional dimensions of trash talk that might be informative, it is possible that a broader typology that solicits ratings on other topics such as “how a person talks” or “how a person smells”—a variable known to have important evolutionary relevance (e.g., Little et al. 2011)—could yield valuable insights. Additionally, although our preliminary study engaged a relatively wide range of sports, future research would gain precision by focusing on trash talk in one or two sports given that “sports” is not a monolithic domain (e.g., Cotton et al. 2011) and that there are substantive differences in potentially important aspects, such as the degree to which members of a team are accountable to each other during the course of actual play.

Taking a step back to consider the broader context of trash-talking, future research could also profitably explore the degree to which effects are elicited by trash-talking. For example, the current research is not designed to examine whether (*a*) there are any effects, (*b*) the trash-talker tends to be affected by the trash talk, and (*c*) the target of the trash-talking tends to be affected (either negatively, as intended, or positively, as suggested by Yip et al.’s (2018) finding that talking trash can help elicit greater creativity). Building on the baseline findings that the current study presents, if one were to assume that trash-talking is reflective of an underlying adaptation (e.g., dispositional traits related to competitiveness) and has been retained as a convention in at least some circumstances because it tends to yield relative advantages for its speakers, then one would reasonably expect that it does have some effects. More specifically, knowledge of the extent to which trash-talking affects the speaker and/or the target(s) may also provide insights into why trash-talking and any related dispositional traits might have been selected over time. For example, such a study would help address whether trash-talking has generally served individuals’ interests to build confidence (as a speaker) or to defend one’s relative standing (in response, as a target).

Just as one might expect variation among individuals with respect to the communication of trash-talking, there is also reason to expect variation among individuals with respect to being influenced by “receiving” such talk. For example, consistent with popular descriptions of people being variably “thin”- or “thick”-skinned in response to insults and criticisms, it is reasonable to expect that some people show more resilience than others—and further, that such resilience can be cultivated (as reviewed by Hinduja and Patchin 2017 for contexts outside of sports). To broaden the context beyond individual differences on dimensions such as resilience, and in light of research suggesting that sports participation by men, at least, is a form of courtship display (e.g., Apostolou and Lambrianou 2017), it might be that the same people who are most likely to trash-talk are also most likely to be responsive to it.

As a final point, just as research involving gossip is sensitive to the fact that it can be employed by members of groups to mob or harass individuals (as reviewed in Kniffin and Wilson 2010), the same is true for trash-talking. Specifically, although our study focuses on describing and understanding the nature of trash-talking in athletic contests, such a study would or should be impossible to complete in contemporary workplace settings where such communications are inappropriate. Indeed, just as personnel departments have obligations to guard against harassment in the workplace, the ways in which referees (whose functions are partly analogous to personnel officers) across different sports penalize players for engaging in certain kinds of trash-talking would be an interesting research topic. In fact, while there are traditions of *celebrating* some degree of trash-talking in sports (contrary to formal analyses that would recognize trash-talking as a definitional aspect of unsporting behavior; Shields et al. 2007), there are certainly examples where players have been penalized for the use of language or taunts that are regarded as “crossing a line” (e.g., Associated Press 2014).

## Conclusion

Across domains, trash-talking is a part of communications between rivals. Indeed, chief executive officers of major global companies are renowned for engaging in it (e.g., Legere 2017), yet the nature of trash-talking has not been previously examined through an evolutionary lens. Our approach, which contends that trash-talking is a kind of communication that anthropologists would more commonly consider to be negative gossip, provides a baseline upon which future research can build. More generally, our focus on trash-talking highlights the fact that competition in sports involves more than just the physical contests among participants.
